# Intestinal Diseases of Children

**Published:** 1901-07

**Authors:** 


### Abstracts.

### INTESTINAL DISEASES OF CHILDREN.

Dentition, in my opinion (George Howard Thompson, M.D., Prof. Mat. Med., etc., St. Louis College of P. and S.), is only incidental to the gastro-intestinal disturbances known under the general name of cholera infantum. Cholera infantum coincidental with teething is apt to be regarded as a result of a physiological process by the thoughtless observer. We must differentiate between a coincidence and a cause. Cholera infantum may take place at any season of the year; so may the process of teething. These two conditions frequently take place simultaneously. Cholera infantum, however, is far more likely to occur in the summer—in the central and southern sections of the United States, in August and September—than in any other season of the year. Indiscretions in diet are the exciting causes. When teething is coincidental we can more readily understand the influence of indiscretion than at a later period. The mere fact of dentition can be responsible only for certain reflex nervous disturbances; but the cause of dentition is the necessity for a change in the child's diet. The child must be supplied with food different from the mother's milk, otherwise, why the advent of teeth? This necessity is recognised intuitively by the mother, who not knowing how to meet the indication physiologically, is only too apt to kill the child with mistaken kindness. The child gets bread to bite, which its gastro-intestinal apparatus is not ready to dispose of properly. It ferments and sets up a gastrointestinal irritation, nausea, vomiting and purging results, and, finally, the doctor is called.

This summer, I have had a large number of cases of cholera infantum, subacute gastrointestinal indigestion and marasmus. Probably my best results were derived from the following line of treatment: Baby was given podophyllin one-fortieth of a grain, or calomel, one-tenth of a grain, until the passages indicated the presence of bile. Then an intestinal antiseptic sedative and astringent consisting of:

| | | |
|---|---|---|
| ℞ | Bismuth subcarbonatis . . . . . . . . . . . . . . . | dr. i. |
| | Glyco-thymoline (Kress) . . . . . . . . . . . . . . . | fl. oz. ss. |
| | Mist. cretæ . . . . . . . . . . . . . . . . | q. s. ad. fl. oz. iij. |

M. Sig. Shake the bottle and take a teaspoonful every three hours.

It is always a good idea to give the baby a enema of a pint of warm water, in which is an ounce of glyco-thymoline at the commencement of treatment, in order that the colon may be relieved as rapidly as possible of its fermenting and decomposing contents, and the lining meet the antiseptic and healing application as soon as possible. Daily enemata of the above solution are frequently advisable.

This remedy is antiseptic; it is nontoxic in the dose given; it is nonirritant; it is healing. It is about the only antiseptic that can be safely given to children.

